# Downbeat Nystagmus Induced by Sedation in Lasik

**DOI:** 10.1155/2012/171679

**Published:** 2012-03-28

**Authors:** Miguel Paciuc-Beja, Gerardo Mendieta

**Affiliations:** ^1^Denver Health Medical Center, Davis Pavillion, Eye clinic, 777 Bannock Street, Denver, Co 80204, USA; ^2^Sanatorio Oftalmologico Merida, Departamento de Anestesia, Chihuahua 71 Col Roma, Mexico City 06700, Mexico

## Abstract

Nystagmus was elicited during lasik under sedation in two patients that were treated for depression. Nystagmus was not present before or after surgery. Nystagmus can be pharmacologically induced and can be a hazard to refractive surgery.

## 1. Introduction

Anterior-segment surgery in adults is usually performed under topical or local anesthesia.

In many of these procedures, the patient is sedated to ease stress and enhance cooperation. 

Lasik is performed under topical anesthesia; nonetheless, some very stress-prone patients may benefit from sedation. Sedation takes away stress, but keeps patients's cooperation intact so they can maintain fixation.

We present the occurrence of nystagmus in a patient undergoing lasik under sedation.

## 2. Case 1

A 45-year-old female refered by her psychiatrist presented myopia of −4.00 sph OU. She presented normal eye movements, no strabismus, and no nystagmus. 

She was stressed thinking about the procedure but wanted to do it, and asked if sedation was possible. She was taking 20 mg of paroxetine per day for 18 months for depression and was doing fine according to the refering psychiatrist. There was no history of neurological disease.

Midazolam and fentanyl were administered prior to surgery by one of us (GM). The flap was lifted uneventfully. When the patient was asked to fixate the red light, a slow low amplitude downbeat nystagmus appeared. With the tracking on, the globe was fixated with the suction ring under low suction. The treatment was completed. The second eye also with nystagmus was treated as the first one.

On the first postoperative day visual acuity was 20/30 OD and 20/25 OS, with no nystagmus upon fixation.

Corneal ablation was centered as shown on corneal topography 45 days after lasik.

## 3. Case 2

A 32-year-old female who was being treated for depression with 20 mg of fluoxetine per day wanted to have refractive surgery. She presented a −3.25 sph OD and −3.75 = −0.75X0 OS. There is no history of neurological disease. She presented normal eye movements, no strabismus, and no nystagmus preoperatively.

Sedation with midazolam and fentanyl was administered prior to surgery.

Upon fixation, a slow downbeat nystagmus was present and surgery was performed without complications. Fixation was maintaind with the suction ring under low suction.

On the first postoperative day, visual acuity was 20/25 OU. No nystagmus was present.

One month after lasik, a corneal topography showed well centered corneal ablations.

## 4. Discussion

Lasik is a painless outpatient surgical procedure performed under topical anesthesia that needs a minimum amount of cooperation from the patient.

Although the great majority of patients cooperate during the procedure, some very stressed patients can benefit by some amount of sedation.

We do not give patients oral tranquilizers prior to any surgical procedure, the reason is variability of action from patient to patient: from the deeply sedated uncooperative patient to the paradoxical effect in some patients with oral tranquilizers that feel more uneasy than before [1]. We use, as in cataract surgery, intravenous sedation.

The usual combination is midazolam and fentanyl, the doses individualized for each patient. Sedation is given by an anesthesiologist (GM).

This is not a routine for lasik patients but only for the very nervous that feel unable to cooperate.

We have been performing lasik with IV sedation since 1998 [2]; this is the first time that nystagmus appeared during the procedure. Pharmacologically induced downbeat nystagmus is triggered by fixation.

These two patients had in common the use of antidepressants (selective serotonin reuptake inhibitors) in addition to sedation with opioids and benzodiazepines. 

Rottach et al. [3] found that after intravenous administration of opiates, eye movements were recorded with the magnetic search coil technique, normal subjects showed a transient disturbance of eye fixation with downbeat nystagmus. Authors hypothesized a cerebellar effect of opiates.

In other ophthalmological surgeries there is no need for the patient to fixate, or they are unable to do it because of the inherent low vision in dense cataracts or retinal surgery. The surgeon manipulates the globe without the need for the patient to fixate a target.

In these cases, the use of antidepressants in combination with opioids (fentanyl) and or benzodiazepines (midazolam) could have contributed to the downbeat nystagmus.

This finding made us aware of the potential hazard of some neuropharmacological agents used by the patient, when sedation will be administered, and we expect a cooperative, fixating patient, as in refractive surgery.
